# Synergistic La/F Co-Doping for Enhanced Lattice Oxygen Stability and Long-Term Cycling Stability in High-Capacity Li-Rich Layered Cathodes

**DOI:** 10.3390/nano16211346

**Published:** 2026-10-23

**Authors:** Jiawei Chen, Zean Yang, Zhihao Zhao, Wenyuan Zhang

**Affiliations:** Laboratory of Materials Design and Quantum Simulation, School of Materials Science and Engineering, Changchun University, Changchun 130022, China; 15354781999@163.com (J.C.); 15592153920@163.com (Z.Y.); zhaozhihao18@163.com (Z.Z.)

**Keywords:** lithium-rich layered oxides, La/F co-doping, lattice oxygen stabilization, cathode materials, lithium-ion batteries

## Abstract

Lithium-rich layered oxides (LLOs) constitute promising cathode candidates for next-generation Li-ion batteries, since their high reversible capacities arise from the combined contribution of transition-metal cations and lattice oxygen redox. However, their practical application is hampered by structural degradation arising from metastable anionic redox. Herein, a synergistic La/F co-doping strategy is reported by us so that the structural and electrochemical stability of LLOs can be enhanced. The complementary effects of La and F co-doping modification and their influence on crystal structure, elemental distribution, and electrochemical performance are systematically investigated. We demonstrate that La/F co-doping stabilizes the oxygen lattice framework, suppresses transition-metal migration into lithium layers, and promotes long-range structural ordering. Mechanistically, La^3+^ doping enlarges the lattice spacing to facilitate Li^+^ diffusion, while F^−^ substitution strengthens metal–fluorine bonds, reinforcing the anionic framework and suppressing irreversible oxygen release. As a result, the La/F co-doped material (LMCNOLF) exhibits markedly improved initial Coulombic efficiency, superior rate capability, and excellent long-term cycling stability. Moreover, it retains 89% of its initial capacity after 300 cycles and outperforms both the La-doped and undoped counterparts. Kinetic analysis reveals that LMCNOLF maintains lower charge-transfer impedance and higher Li^+^ diffusion coefficients. This work elucidates the mechanism of La/F synergistic doping in stabilizing lattice oxygen and suppressing structural degradation, offering a rational design route for high-performance Li-rich layered cathodes.

## 1. Introduction

Lithium-ion batteries combining high energy density, long cycle life, and superior safety are urgently needed. This demand is driven by the global energy transition as well as the rapid proliferation of electric vehicles and portable electronic devices [1,2,3]. However, conventional intercalation cathodes are intrinsically constrained by a single transition-metal cation redox mechanism and typically deliver a reversible specific capacity below 200 mAh g^−1^. This limitation has become a critical bottleneck impeding further improvements in battery energy density [4,5]. In contrast, lithium-rich layered oxides (LLOs) can deliver a high reversible specific capacity exceeding 250 mAh g^−1^ through a dual redox mechanism involving both transition-metal cations and lattice oxygen anions [6,7,8]. In particular, at high voltages above 4.5 V, the monoclinic Li_2_MnO_3_ component undergoes electrochemical activation, accompanied by lattice oxygen oxidation and excessive lithium-ion extraction [9]. This process breaks the traditional theoretical capacity limit and offers the prospect of increasing battery energy density by more than 30%, positioning LLOs as one of the most promising candidates for next-generation cathodes [10,11].

Despite the increasing maturity of their synthesis processes, the commercialization of lithium-rich cathodes remains severely restricted by three intrinsic defects: first, a low initial Coulombic efficiency (ICE) of 60–80% [12], caused by irreversible oxygen evolution and interfacial side reactions during the first cycle; second, poor rate capability arising from sluggish electronic conductivity and Li^+^ diffusion; and third, continuous voltage decay during prolonged cycling [13,14,15,16]. This decay originates primarily from the metastable nature of lattice oxygen, which triggers irreversible oxygen release, transition-metal migration into lithium layers, and a layered-to-spinel phase transformation upon extended cycling [17]. Stabilizing the lattice oxygen framework is therefore critical to mitigating the structural degradation of lithium-rich cathodes [18,19,20].

Extensive efforts have been devoted to mitigating the structural degradation of lithium-rich cathodes. For instance, Wang et al. adopted bulk doping with high-bond-energy elements to regulate the oxygen electronic state and reinforce the crystal structure, which partially alleviated the degradation [21]. However, this strategy offers limited improvement in lattice oxygen stability and may introduce additional structural stress [22,23]. Similarly, Zhang et al. focused on surface/interface engineering by constructing coating layers or heterojunctions to block electrolyte corrosion and inhibit surface oxygen loss, achieving favorable results [24]. Nevertheless, such surface modifications can hardly resolve the metastable nature of bulk lattice oxygen [25], and challenges remain in controlling coating thickness and maintaining interfacial stability [26,27,28]. More importantly, most reported studies rely on a single regulation route, lacking a synergistic optimization of both bulk oxygen lattice stability and interfacial structural integrity, which makes long-term suppression of degradation difficult to achieve [29,30,31]. In addition, the dynamic evolution of lattice oxygen during cycling and its intrinsic coupling with structural degradation have not been fully elucidated, thereby limiting the rational design of highly stable lithium-rich cathode materials [32,33,34,35].

To address these challenges, this study takes lattice oxygen stabilization as the core concept and proposes a synergistic La/F co-doping strategy for lithium-rich layered cathodes. We systematically investigate the combined effects of La/F co-doping on the crystal structure, surface chemical states, elemental distribution, and electrochemical performance of the material, thereby clarifying the mechanism by which La/F co-doping stabilizes the lattice oxygen framework. This work aims to stabilize the oxygen lattice framework, suppress cation mixing, and enhance the long-range ordering of the layered structure through synergistic La/F co-doping, ultimately leading to markedly improved initial Coulombic efficiency, rate capability, and long-term cycling stability. This study is expected to offer robust experimental evidence and theoretical guidance, so that high-capacity Li-rich layered cathodes with enhanced structural and electrochemical stability can be rationally designed.

## 2. Materials and Methods

### 2.1. Materials and Synthesis

In this work, a La-F co-doped lithium-rich layered oxide cathode material, Li_1.25_Mn_0.52_Ni_0.125_Co_0.125_La_0.01_F_0.015_O_2_, denoted as LMCNOLF, was prepared via a sol–gel method. Concurrently, the La-doped Li_1.25_Mn_0.52_Ni_0.125_Co_0.125_La_0.01_O_2_, F-doped Li_1.25_Mn_0.52_Ni_0.125_Co_0.125_F_0.015_O_2_ and undoped Li_1.25_Mn_0.52_Ni_0.125_Co_0.125_O_2_, denoted as LMCNOL, LMCNOF and LMCNO, were synthesized using exactly the same procedure as control samples. Stoichiometric amounts of Mn(CH_3_COO)_2_·4H_2_O, Ni(CH_3_COO)_2_·4H_2_O, Co(CH_3_COO)_2_·4H_2_O, La(CH_3_COO)_3_·6H_2_O, and LiF were accurately weighed as the metal and doping sources, with citric acid monohydrate employed as the chelating agent. All the reagents were added into 25 mL of deionized water and fully dissolved via magnetic stirring to yield a homogeneous mixed solution. Subsequently, we introduced an 8% excess of Li_2_CO_3_ as the lithium source and continuously stirred the mixture for 10 min so that all components could be uniformly dispersed. Under constant stirring at 500 rpm, aqueous ammonia was slowly added dropwise into the solution to precisely adjust the pH to 8.5 ± 0.1, maintaining a homogeneous and precipitate-free solution throughout the entire process. The as-prepared precursor solution was transferred to a constant-temperature water bath at 85 °C, and evaporated under magnetic stirring for 7–8 h until complete volatilization of water to form a viscous purple gel. After being transferred to a vacuum drying oven, the obtained gel was vacuum dried at 150 °C for 12 h. After thorough grinding, the dried gel was placed in a muffle furnace under an air atmosphere. First, pre-calcination was conducted at 450 °C for 6 h to complete the decomposition of organic matter and preliminary decomposition of the precursor. The temperature was subsequently elevated to 900 °C and held for 12 h, thereby facilitating crystallization of the material. After naturally cooling along with the furnace, bulky calcined product was obtained. After thorough grinding into fine powder, the bulky calcined products were washed sequentially with absolute ethanol and deionized water for multiple cycles, so that surface lithium salt impurities generated during the calcination process could be removed. Following the washing step, drying of the powder was carried out in a vacuum drying oven at 80 °C for 12 h, after which the target lithium-rich layered cathode materials were finally obtained.

### 2.2. Characterization

To systematically characterize the phase composition, crystalline structure, surface morphology, and chemical states of the as-prepared materials, multiple complementary characterization techniques were employed. Powder X-ray diffraction (XRD) measurements were performed on a Bruker AXS D8 Advance diffractometer, which operated at 40 kV and 40 mA with Cu Kα radiation (λ = 1.5406 Å). The data were collected in the 2θ range of 10.0–80.0°. A step increment of 0.1° was used throughout the measurement. Field-emission scanning electron microscopy (FE-SEM, JEOL JSM-7500F), operated at an accelerating voltage of 10 kV, was employed for the analysis of microstructural morphology and elemental distribution, and it was coupled with energy-dispersive X-ray spectroscopy (EDS), by which qualitative elemental distribution mapping was enabled. X-ray photoelectron spectroscopy (XPS, Thermo Scientific K-Alpha) was employed for material characterization. Specifically, the surface chemical composition and oxidation states of the synthesized materials were determined.

### 2.3. Electrochemical Measurements

CR2025-type coin cells were fabricated for electrochemical evaluation, by which the properties of the electrode materials were assessed. A homogeneous slurry, which contained active material, conductive carbon, and polyvinylidene fluoride (PVDF) binder in a mass ratio of 8:1:1, was uniformly cast onto aluminum foil to prepare working electrodes. The coated foil was first vacuum-dried at 80 °C. It was then punched into circular electrodes with a diameter of 12 mm. Half-cells were subsequently fabricated. Lithium metal was used as the counter electrode, a Celgard-2500 microporous membrane served as the separator, and the electrolyte was 1.0 M LiPF_6_ in ethylene carbonate/diethyl carbonate (EC/DEC, 1:1 by volume) with 10 vol% fluoroethylene carbonate (FEC). Room-temperature charge–discharge tests within the voltage window of 2–4.8 V were conducted on a LAND CT2001A battery tester. The Coulombic efficiency was calculated as the percentage ratio of the discharge specific capacity to the corresponding charge specific capacity in each cycle. For galvanostatic intermittent titration technique (GITT) analysis, we employed the same apparatus, in which an initial activation cycle at 10 mA g^−1^ was performed first, followed by alternating 30-min current pulses and 4-h open-circuit relaxation steps. Finally, we measured EIS on a Zahner workstation, which covered a frequency range of 0.01 Hz to 100 kHz with a 5 mV AC amplitude perturbation.

## 3. Results and Discussion

**Figure 1:**
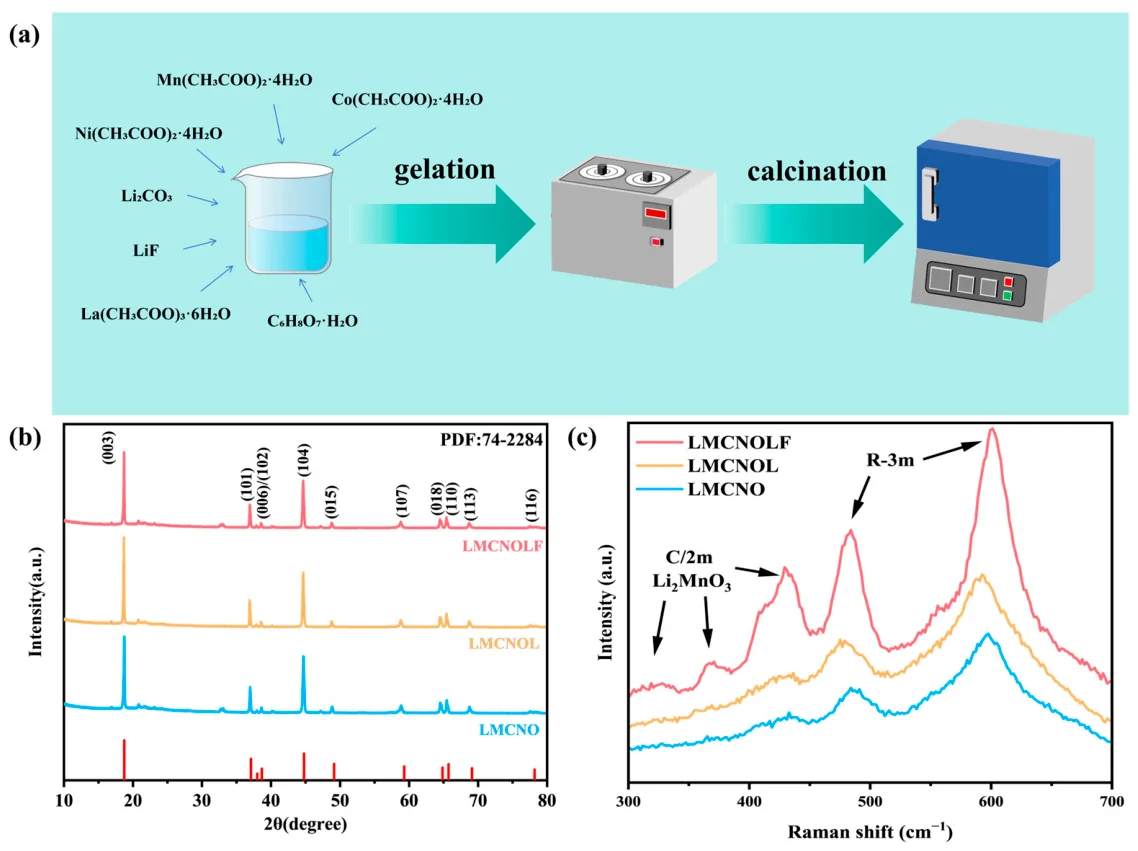
Synthesis route and structural characterization of LMCNO, LMCNOL, and LMCNOLF. (**a**) Schematic of the sol–gel synthesis process involving gelation and calcination. (**b**) XRD patterns of the three samples (JCPDS No. 74-2284). (**c**) Raman spectra of the three samples.

A sol–gel method was employed to synthesize La/F co-doped Li-rich layered oxide cathode materials, as depicted in Figure 1a. XRD and Raman spectroscopy were employed to characterize the phase composition, crystalline structure, and chemical bonding states of the three as-prepared samples: undoped LMCNO, La-doped LMCNOL, and La/F co-doped LMCNOLF. The corresponding XRD results are presented in Figure 1b. These results demonstrate that the main diffraction peaks of all three samples are assignable to a hexagonal α-NaFeO_2_-type layered structure with the R-3m space group. Weak superlattice diffraction peaks that appear in the 2θ range of 20–25° are assigned to the monoclinic Li_2_MnO_3_ phase with the C2/m space group. Their relatively weak intensity is typical for these materials and indicates that the typical Li-rich solid-solution structure consisting of a rhombohedral layered main phase and a monoclinic Li_2_MnO_3_ phase is successfully preserved in all samples. No impurity phase peaks are observed in the XRD patterns, indi cating that La/F doping does not introduce secondary phases and that the dopant ions are incorporated into the crystal lattice without disrupting the host phase.

Proceeding from the undoped LMCNO to the La-doped LMCNOL and La/F co-doped LMCNOLF, the intensity ratio of the (003) to (104) planes, I(003)/I(104), gradually increases, while the splitting of the doublet peaks at (006)/(102) and (018)/(110) becomes more pronounced. These specific peak variations are highly significant, providing indirect macroscopic evidence that our doping strategy effectively mitigates Li/transition-metal cation mixing and enhances the long-range ordering of the layered framework. In addition, the doped samples exhibit a slight shift in the diffraction peaks toward lower 2θ angles (Figure S3), which arises from lattice expansion caused by the incorporation of larger La^3+^ ions at transition-metal sites. This expansion enlarges the interplanar spacing and is expected to facilitate Li^+^ transport during electrochemical cycling. As shown in Figure 1c, the Raman spectra illustrate the structural characteristics of the LMCNO, LMCNOL, and LMCNOLF samples. The strong peaks around 480 and 600 cm^−1^ are assigned to the R-3m layered main phase, while the weak peaks in the 300–450 cm^−1^ region correspond to the C/2m phase. This indicates that all three materials maintain a complete lithium-rich solid solution structure, and doping does not disrupt the main lattice. Crucially, the LMCNOLF sample exhibits the highest Raman signal intensity and the sharpest peak shape. This enhanced signal demonstrates that La/F co-doping significantly improves the crystallinity of the material and promotes the formation of stronger chemical bonds, thereby effectively enhancing the overall structural stability of the material.

**Figure 2:**
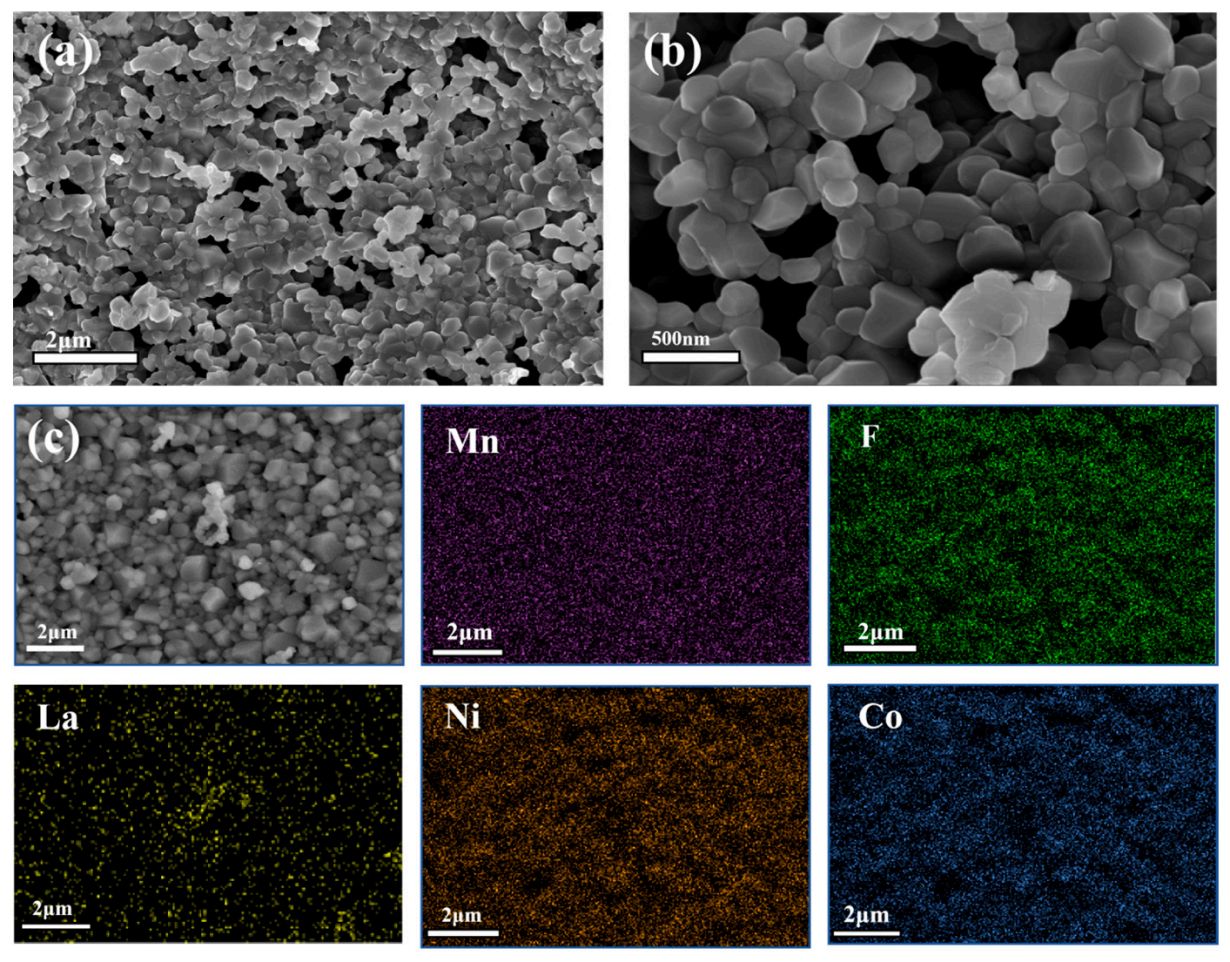
Morphological characterizations of the LMCNOLF: (**a**) low-magnification SEM micrograph; (**b**) high-magnification SEM micrograph; (**c**) low-magnification SEM micrograph and corresponding EDS elemental mappings of Mn, F, La, Ni, and Co.

Figure 2 presents the SEM morphologies and EDS elemental mapping results of the LMCNOLF sample. Figure 2a,b, Figures S1 and S2 show the particle morphologies at different magnifications, while Figure 2c presents the morphological image of the corresponding region together with the elemental mappings of Mn, Ni, Co, La, and F. As revealed by the low-magnification SEM image (Figure 2a), the sample consists of numerous aggregated submicron-scale particles with a relatively uniform size distribution and slight agglomeration, which is typical of particle sintering and growth during high-temperature calcination. All particles exhibit clear contours without obvious amorphous impurities or abnormally large grains, indicating good crystallinity and a pure phase composition.

The high-magnification SEM image (Figure 2b) reveals the morphology of the primary particles. These particles are near-spherical to polygonal, and their sizes are mainly distributed in the 200–500 nm range. The particles have smooth surfaces and well-defined grain boundaries, indicating high crystallinity. The solid-state diffusion distance of Li^+^ can be shortened and the electrode–electrolyte contact area enlarged by such particle sizes, which is advantageous for rate capability and reaction kinetics. The EDS elemental mappings (Figure 2c) show that Mn, Ni, and Co are homogeneously distributed across the mapping region without local enrichment or segregation. The dopants La and F exhibit a similar distribution, matching that of the matrix elements, with no detectable aggregation or surface segregation. These results strongly indicate that La^3+^ and F^−^ are incorporated into the bulk lattice and uniformly dispersed rather than merely adsorbed on the particle surfaces, fully confirming that the proposed sol–gel La/F co-doping process is highly efficient and successful. To further investigate the porous characteristics and specific surface area of the materials, nitrogen adsorption and desorption measurements were conducted. As shown in Figure S4, the isotherms exhibit a typical Type IV curve with a distinct hysteresis loop, indicating the presence of a mesoporous architecture formed by the aggregation of submicron primary particles. The corresponding pore size distribution derived from the desorption branch confirms that the pores are predominantly distributed in the mesoporous range. This well-defined mesoporous structure is highly advantageous, as it significantly increases the effective electrode–electrolyte contact area, facilitates efficient electrolyte penetration, and provides abundant pathways to accelerate lithium ion transport kinetics during prolonged electrochemical cycling.

**Figure 3:**
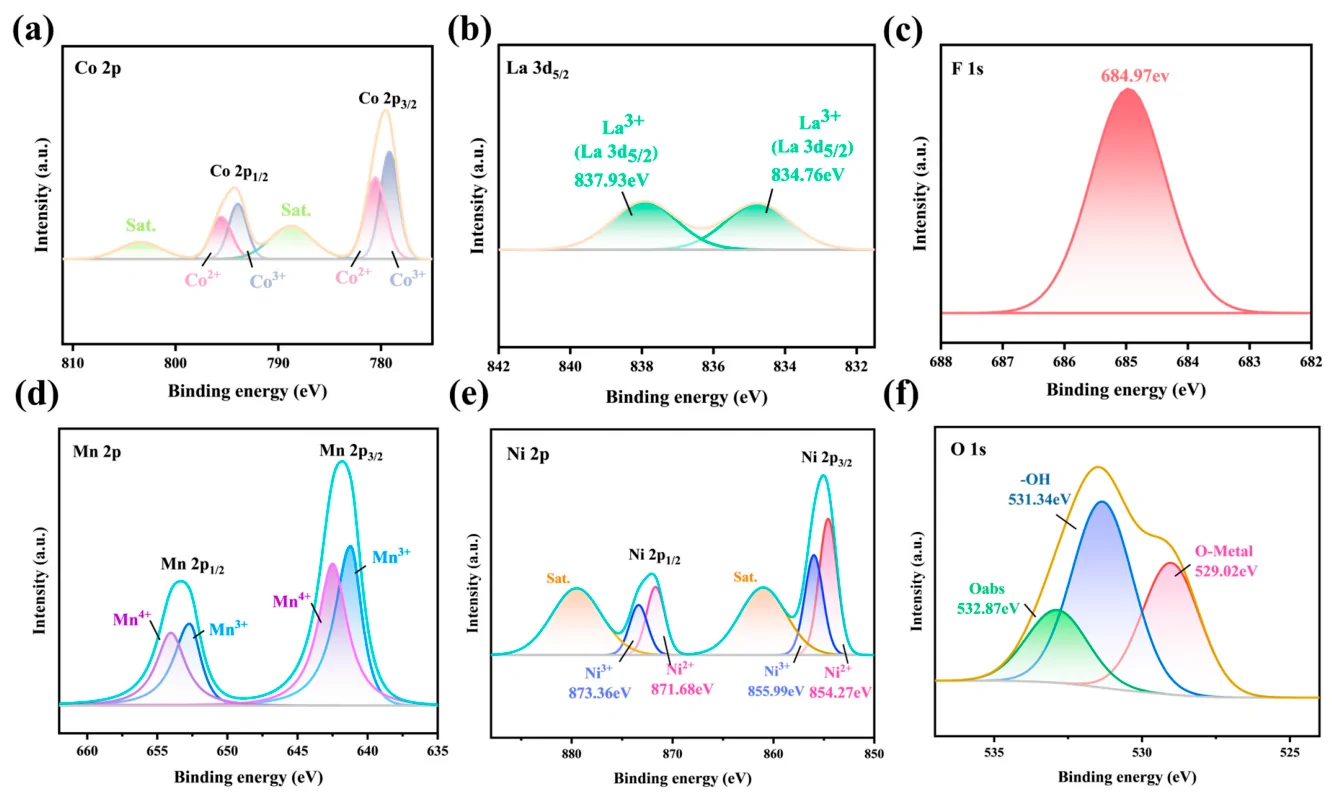
XPS characterizations of LMCNOLF: (**a**) fitted Co 2p XPS spectrum; (**b**) fitted La 3d_5_/_2_ XPS spectrum; (**c**) fitted F 1s XPS spectrum; (**d**) fitted Mn 2p XPS spectrum; (**e**) fitted Ni 2p XPS spectrum; (**f**) fitted O 1s XPS spectrum.

So that the effectiveness of doping might be verified and the resulting electronic structure modulation revealed, the surface chemical states and bonding environments of the La/F co-doped Li-rich layered oxide cathode were characterized by means of high-resolution XPS (Figure 3). The Co 2p, Mn 2p, and Ni 2p spectra all exhibit characteristic spin–orbit splitting features. Peak fitting indicates that Co and Ni coexist in mixed +2/+3 valence states, while Mn adopts a mixed +3/+4 configuration, consistent with the intrinsic transition-metal valence states of Li-rich layered oxides. Deconvolution of the La 3d_5_/_2_ spectrum yields two components centered at 837.93 and 834.76 eV, both of which are attributable to La^3+^, thereby indicating that La is incorporated into the host lattice in the +3 oxidation state. The F 1s spectrum exhibits a single symmetric peak at 684.97 eV, corresponding to metal–fluorine bonds, indicating that F is incorporated into the oxygen sublattice as F^−^ and is expected to reinforce the anionic framework through the formation of high-bond-energy M–F bonds. Resolution of the O 1s spectrum gives three components, which are lattice oxygen, hydroxyl oxygen, and defect-adsorbed oxygen. This allows the lattice oxygen fraction and oxygen vacancy concentration to be quantified, providing a surface chemical basis for evaluating lattice oxygen stability. Taken together, these structural and chemical characterization results indicate that La/F co-doping preserves the layered host structure, promotes long-range structural ordering, and incorporates both dopants uniformly into the lattice, thereby stabilizing the oxygen sublattice and providing a structural basis for the enhanced electrochemical performance discussed below.

**Figure 4:**
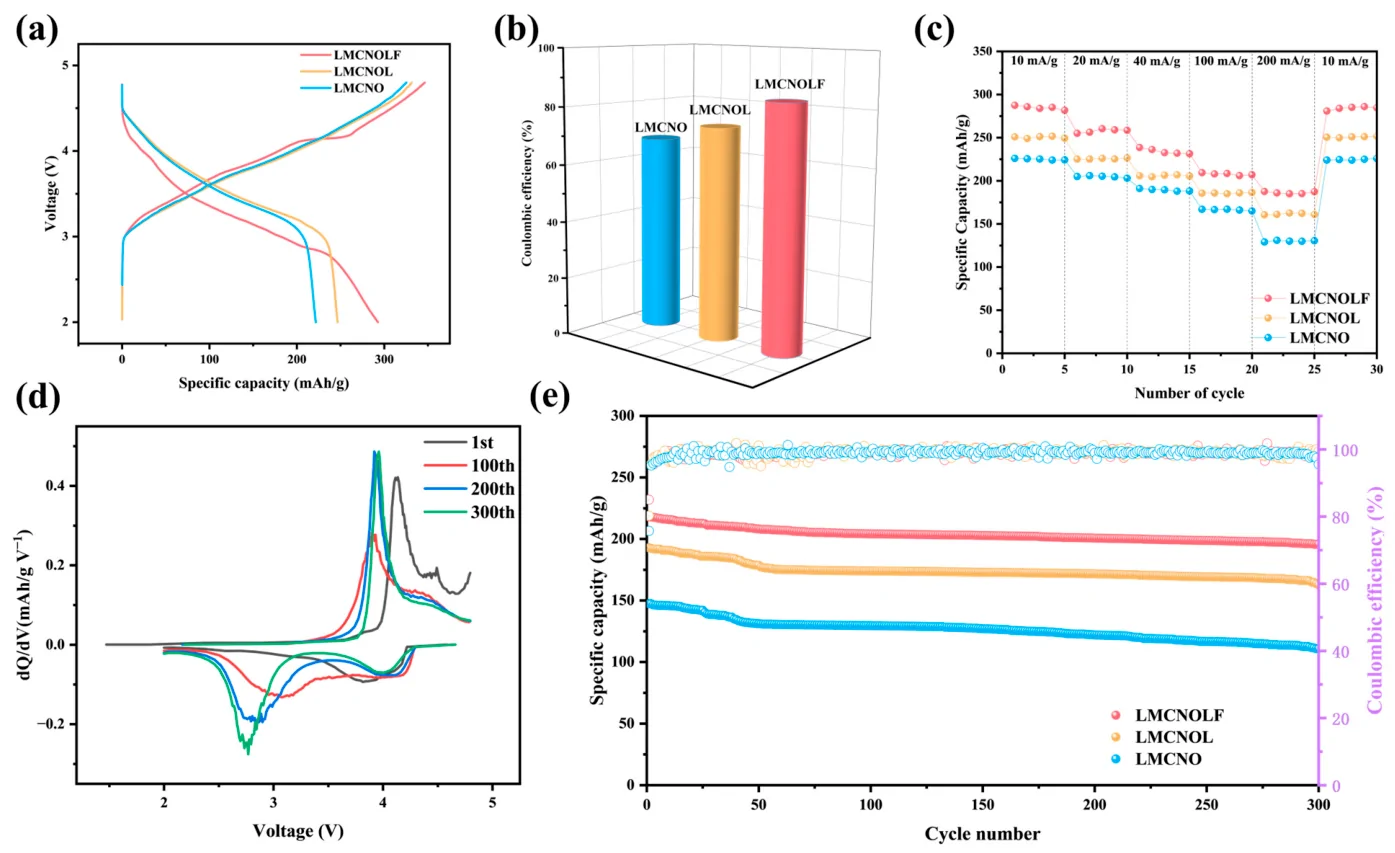
Electrochemical characterization of LMCNO, LMCNOL, and LMCNOLF samples: (**a**) initial galvanostatic charge–discharge curves; (**b**) comparison of initial Coulombic efficiency; (**c**) rate performance measured under stepwise varied current densities; (**d**) dQ/dV differential capacity curves at the 1st, 100th, 200th and 300th cycles; (**e**) long-term cycling stability and corresponding Coulombic efficiency.

In order to determine whether the structural stabilization induced by La/F co-doping translates into electrochemical benefits, the electrochemical performance of the three samples was next assessed. The first charge–discharge curves of the three samples at 0.1 C are displayed in Figure 4a, while the corresponding initial Coulombic efficiencies are presented in Figure 4b. All samples exhibit the typical two-stage charging behavior of Li-rich layered oxides. The electrochemical reaction of the R-3m layered main phase is reflected in the sloping voltage region below 4.5 V, in which Ni^2+^/Ni^4+^ and Co^3+^/Co^4+^ are oxidized and Li^+^ is extracted from the lithium layer. The electrochemical activation of the monoclinic C2/m Li_2_MnO_3_ phase is reflected in the long voltage plateau above 4.5 V, during which lattice oxygen anions are oxidized and excess Li^+^ in the transition-metal layer is extracted, thereby accounting for the ultrahigh specific capacity of Li-rich materials. The gradual reduction of transition-metal cations and the intercalation of Li^+^ are reflected in the discharge process, which appears as a continuous sloping curve.

The initial charge–discharge capacities differ markedly among the three samples. Although the undoped LMCNO delivers a high initial charge capacity of 325 mAh g^−1^, it also shows the lowest initial discharge capacity of 221 mAh g^−1^, suggesting that side reactions such as irreversible lattice oxygen release and electrolyte oxidative decomposition consume a substantial fraction of the charging capacity and thereby lower the reversible capacity ratio. While the discharge capacity rises to 246 mAh g^−1^ after La doping, the La/F co-doped LMCNOLF delivers the highest initial discharge capacity of 292 mAh g^−1^ and an initial charge capacity of 346 mAh g^−1^, with the irreversible capacity loss at the charging plateau being significantly reduced. Correspondingly, the initial Coulombic efficiency increases progressively from 68% for LMCNO to 74% for LMCNOL and further to 84% for LMCNOLF.

To evaluate the rate capability of the three samples, we measured their discharge capacities at different current densities, as shown in Figure 4c. A higher discharge capacity than that of LMCNOL and undoped LMCNO is delivered by LMCNOLF at all current densities, and this advantage is found to become more pronounced as the current density increases. At 200 mA g^−1^, the discharge capacity of undoped LMCNO decreases to approximately 132 mAh g^−1^. LMCNOL maintains around 160 mAh g^−1^, while LMCNOLF still retains over 185 mAh g^−1^. When the current density returns to 10 mA g^−1^, all three samples recover to near their initial capacities, with LMCNOLF showing the highest recovery. This indicates that LMCNOLF does not sustain irreversible structural damage under the strong polarization of high-current cycling and exhibits excellent electrochemical reversibility. The improved rate capability can be attributed to two main mechanisms. First, the larger ionic radius of La^3+^ relative to the transition-metal ions induces lattice expansion when La^3+^ substitutes at transition-metal sites, widening the two-dimensional Li^+^ diffusion channels, lowering the energy barrier for Li^+^ solid-state diffusion, and accelerating ion transport kinetics. Second, F doping reinforces the crystal framework, preserving the integrity of the layered structure during high-current cycling and preventing Li^+^ transport pathways from being blocked by structural collapse. Together with the submicron particle size that shortens the Li^+^ diffusion distance, these factors collectively account for the enhanced rate capability.

The structural evolution and the associated changes in redox behavior during cycling were investigated. For this purpose, dQ/dV curves were derived from the charge–discharge profiles of LMCNOLF at different cycle numbers (Figure 4d). In the first cycle, the strong peak near 4.1 V on the oxidation side corresponds to transition-metal oxidation and Li^+^ extraction from the layered phase, while the weak signal above 4.5 V corresponds to the activation of the Li_2_MnO_3_ phase. The main reduction peak near 3.8 V is sharp and symmetric, indicating low polarization and high reversibility. As cycling proceeds from the 1st to the 300th cycle, the main reduction peak gradually shifts to lower voltages, with slight intensity attenuation and peak broadening. This behavior is typical of Li-rich materials and arises from the progressive layered-to-spinel reconstruction and the increased Li^+^ diffusion barrier caused by transition-metal migration. Nevertheless, after 300 cycles, the main redox peak still retains a well-defined profile, with only a small peak shift and no severe broadening. This mitigated peak polarization provides indirect evidence that La/F co-doping retards the structural phase transition and mitigates the voltage decay rate. This benefit is likely associated with the enhanced anion framework and the reduced cation mixing, which together hinder transition-metal migration and stabilize the layered structure.

The long-term cycling performance and the corresponding Coulombic efficiencies of the three samples over 300 cycles at 100 mA g^−1^, together with the cycling performance of the four samples over 100 cycles in Figure S5, are presented in Figure 4e and Figure S3. In the initial stage of cycling, LMCNOLF delivers the highest discharge capacity, markedly higher than that of LMCNOL, LMCNOF and LMCNO. As cycling proceeds, the capacity of undoped LMCNO decays rapidly, retaining only 74% after 300 cycles, the lowest among the three samples. La doping improves the cycling stability to 84% retention, while LMCNOLF exhibits the slowest capacity decay, maintaining the highest discharge capacity and an optimal capacity retention of 89% after 300 cycles (Table S1). The Coulombic efficiency of all three samples rises rapidly in the initial stage and then stabilizes. Among the three samples, LMCNOLF stabilizes most rapidly and maintains a value close to 100% over extended cycling, indicating a highly reversible electrode reaction and effective suppression of side reactions at the electrode–electrolyte interface. The improved long-term cycling performance arises from the synergistic effect of bulk structural stability and interfacial optimization. At the bulk level, La/F co-doping strengthens the M-O/F bonding and stabilizes the oxygen sublattice, which macroscopic electrochemical evaluations suggest indirectly suppresses transition-metal migration and the layered-to-spinel-to-rock-salt structural reconstruction during cycling. This also alleviates the loss of active material caused by irreversible lattice oxygen release. At the interfacial level, the oxidative decomposition of the electrolyte is decreased by reduced lattice oxygen release, while the catalytic side reactions between dissolved transition metals and the electrolyte are limited by suppressed transition-metal dissolution. The formation of a thin and stable electrode–electrolyte interphase is favored by these effects, which ultimately contributes to stable capacity retention during cycling.

**Figure 5:**
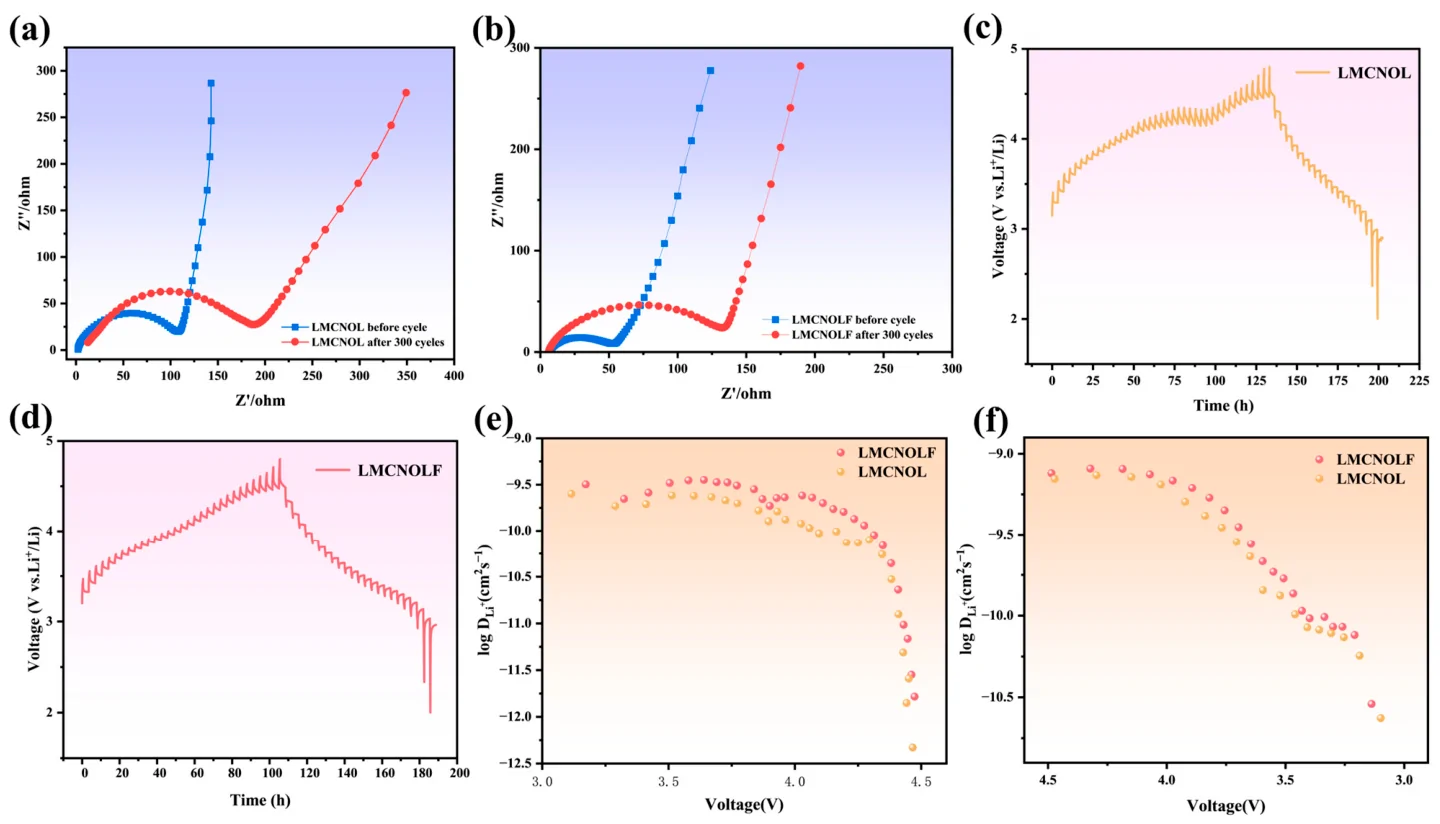
Kinetic analysis of LMCNOL and LMCNOLF electrodes. (**a**,**b**) Nyquist plots of LMCNOL and LMCNOLF before cycling and after 300 cycles. (**c**,**d**) GITT curves of LMCNOL (**c**) and LMCNOLF (**d**). (**e**,**f**) Variation in log (D_Li+_) with voltage for LMCNOLF and LMCNOL during charge (**e**) and discharge (**f**).

As shown in Figure 5a,b and Figure S6, EIS was performed on LMCNO, LMCNOL, and LMCNOLF electrodes before and after 300 cycles to evaluate the evolution of interfacial kinetics and Li^+^ diffusion behavior. All Nyquist plots were fitted using a modified Randles equivalent circuit, R_s_(CPE-R_ct_)Z_w_, where the high-frequency intercept corresponds to the ohmic resistance (R_S_), the depressed semicircle in the high-to-middle frequency region represents the interfacial charge-transfer resistance (R_ct_) coupled with a constant phase element (CPE), and the low-frequency inclined line reflects the Warburg diffusion impedance (Z_w_). Prior to cycling, all three fresh electrodes exhibit small Rs values (3.8–5.1 Ω), while their initial R_ct_ decreases progressively from 143.5 Ω for LMCNO to 104.2 Ω for LMCNOL and 49.3 Ω for LMCNOLF, indicating that La/F co-doping facilitates initial interfacial charge transfer. After 300 cycles, the R_ct_ of pristine LMCNO surges to 292.4 Ω (∆R_ct_ = 148.9 Ω) due to severe electrolyte decomposition and structural degradation, whereas LMCNOL shows a moderated R_ct_ of 178.6 Ω (∆R_ct_ = 74.4 Ω). Notably, LMCNOLF maintains the lowest post-cycling R_ct_ of 125.7 Ω alongside a steep low-frequency Warburg slope, confirming that La/F co-doping effectively suppresses interfacial degradation and phase transformation to preserve rapid charge-transfer capability during long-term cycling.

Figure 5c,d present the GITT profiles of LMCNOLF and LMCNOL, respectively. The Li^+^ solid-phase diffusion coefficient (D_Li_^+^) under different charge–discharge states can be quantified from the periodic galvanostatic pulse and open-circuit relaxation process. In the GITT curves, under the same current pulses, LMCNOLF exhibits smaller voltage polarization and reaches a steady state more rapidly during open-circuit relaxation, indicating superior ion transport kinetics and lower concentration polarization. Calculation of the diffusion coefficients is performed on the basis of Fick’s second law. A linear relationship between the applied constant current and the relaxation time is assumed. On this basis, the formula for the Li^+^ diffusion coefficient (D_Li_^+^) can be simplified as follows:

(1) $$D_{{\mathrm{Li}}^{+}}=\frac{4}{\pi \tau }{\left(\frac{m_{B}V_{M}}{M_{B}S}\right)}^{2}{\left(\frac{{∆E}_{s}}{{∆E}_{\tau }}\right)}^{2}$$

The parameters in this formula are defined as follows. Specifically, m_B_ represents the mass of the active material, while M_B_, V_M_, and S correspond to the molar mass, the molar volume, and the electrode–electrolyte interfacial area, respectively. Figure 5e,f present the Li^+^ diffusion coefficients that were calculated from the GITT curves during charge and discharge. The charging process from 3.0 to 4.5 V is represented by Figure 5e, whereas the discharging process from 4.5 to 3.0 V is represented by Figure 5f. Over the entire voltage range, the D_Li_^+^ values of both samples decrease gradually with increasing voltage, i.e., with deepening delithiation. This decrease is particularly pronounced at high voltages. This phenomenon is related to the decrease in Li^+^ concentration and the local structural distortion at high delithiation states, which together lead to increased diffusion resistance.

Notably, the log(D_Li_^+^) values of LMCNOLF are higher than those of LMCNOL across the entire voltage range, indicating generally faster Li^+^ diffusion, especially above 4.0 V. The improved Li^+^ diffusion originates from two structural effects. First, the two-dimensional Li^+^ diffusion channels in the layered structure are broadened by lattice expansion induced by the larger La^3+^ ions, and the diffusion energy barrier is thereby lowered. Second, F doping enhances the structural stability, preserving the integrity of the diffusion channels during cycling and preventing ion transport pathways from being blocked by structural collapse. Collectively, these two effects accelerate Li^+^ transport within the layered framework, thereby providing a kinetic basis for the enhanced rate capability and long-term cycling stability of LMCNOLF.

## 4. Conclusions

A synergistic La/F co-doping strategy was developed in this study so that the lattice oxygen instability and structural degradation of high-capacity Li-rich layered oxide cathodes could be addressed. Through systematic structural, morphological, and electrochemical characterizations, we found that La^3+^ doping induces lattice expansion and broadens the Li^+^ diffusion channels, while F^−^ doping forms stronger M–F bonds that stabilize the lattice oxygen framework and suppress oxygen release and cation mixing. The La/F co-doped material demonstrates improved initial Coulombic efficiency, enhanced rate capability, and excellent long-term cycling stability. Notably, 89% of its initial capacity is retained after 300 cycles. Kinetic analysis further reveals lower charge-transfer impedance and enhanced Li^+^ diffusion kinetics for LMCNOLF. This work provides insight into how La/F co-doping stabilizes lattice oxygen and suppresses structural degradation. Furthermore, it offers experimental evidence and guidance for the design of high-performance Li-rich layered cathodes, which possess enhanced structural stability.

## Data Availability

The original contributions presented in this study are included in the article/Supplementary Materials. Further inquiries can be directed to the corresponding author.

## Supplementary Materials

The following supporting information can be downloaded at https://www.mdpi.com/article/10.3390/nano16211346/s1: Figure S1. SEM image of LMCNOLF, showing primary crystalline particles with scale bar of 500 nm. Figure S2. SEM image of LMCNOL material with a scale bar of 500 nm. Figure S3. Locally magnified XRD patterns of the (003) diffraction peak for LMCNO, LMCNOL, and LMCNOLF samples (indexed to JCPDS/PDF No. 74-2284), illustrating the peak shift toward lower 2θ angles upon doping. Figure S4. Nitrogen adsorption–desorption isotherms of the synthesized sample. The inset shows the corresponding pore size distribution curve calculated from the desorption branch. Figure S5. Variation of specific capacity versus cycle number for LMCNO, LMCNOL, LMCNOF and LMCNOLF cathodes over 100 cycles. Figure S6. EIS Nyquist spectra of LMCNO before cycling and after 300 cycles. Figure S7. Equivalent electrical circuit model (Rs(CPE-Rct)Zw) used for fitting the electrochemical impedance spectra of the LMCNO, LMCNOL, and LMCNOLF electrodes. Figure S8. Energy-dispersive X-ray spectroscopy (EDS) spectrum of the LMCNOLF sample, confirming the presence of Mn, Ni, Co, La, and F elements. Table S1. Summary of electrochemical performance parameters for the LMCNO, LMCNOL, and LMCNOLF cathode electrodes.
