# Supplementary material for: Synergistic La/F Co-Doping for Enhanced Lattice Oxygen Stability and Long-Term Cycling Stability in High-Capacity Li-Rich Layered Cathodes
Source: Nanomaterials (Basel). 2026 Oct 23;16(21):1346. doi: 10.3390/nano16211346 (PMC13764899; doi:10.3390/nano16211346)
Supplement: Supplementary file 1 [file nanomaterials-16-01346-s001.zip › nanomaterials-4621501-supplementary.pdf]

Supporting Information

Synergistic La/F Co-Doping for Enhanced Lattice Oxygen Stability and Long-Term Cycling Stability in High-Capacity Li-Rich Layered Cathodes

Jiawei Chen, Zean Yang, Zhihao Zhao and Wenyuan Zhang *

Laboratory of Materials Design and Quantum Simulation, School of Materials Science and Engineering, Changchun University, Changchun 130022, China; 15354781999@163.com (J.C.); 15592153920@163.com (Z.Y.); zhaozhihao18@163.com (Z.Z.)

* Correspondence: zhangwy@ccu.edu.cn

Table of contents:

Figure S1. SEM image of LMCNOLF, showing primary crystalline particles with scale bar of 500 nm.

Figure S2. SEM image of LMCNOL material with a scale bar of 500 nm.

Figure S3. Locally magnified XRD patterns of the (003) diffraction peak for LMCNO, LMCNOL, and LMCNOLF samples (indexed to JCPDS/PDF No. 74-2284), illustrating the peak shift toward lower 2θ angles upon doping.

Figure S4. Electrochemical performance of the single F-doped sample (LMCNOF) alongside LMCNO, LMCNOL, and LMCNOLF: (a) Initial galvanostatic charge-discharge curve of LMCNOF at 10 mA g^{-1}.; (b) Long-term cycling performance of LMCNO, LMCNOL, LMCNOF, and LMCNOLF cathodes over 100 cycles at 100 mA g^{-1}.

Figure S5. EIS Nyquist spectra of LMCNO before cycling and after 300 cycles.

Figure S6. Equivalent electrical circuit model (R_s(CPE-R_{ct})Z_w) used for fitting the electrochemical impedance spectra of the LMCNO, LMCNOL, and LMCNOLF electrodes.

Figure S7. Energy-dispersive X-ray spectroscopy (EDS) spectrum of the LMCNOLF sample, confirming the presence of Mn, Ni, Co, La, and F elements.

Figure S8. Energy-dispersive X-ray spectroscopy (EDS) spectrum of the LMCNOLF sample, confirming the presence of Mn, Ni, Co, La, and F elements.

Table S1. Summary of electrochemical performance parameters for the LMCNO, LMCNOL, and LMCNOLF cathode electrodes.

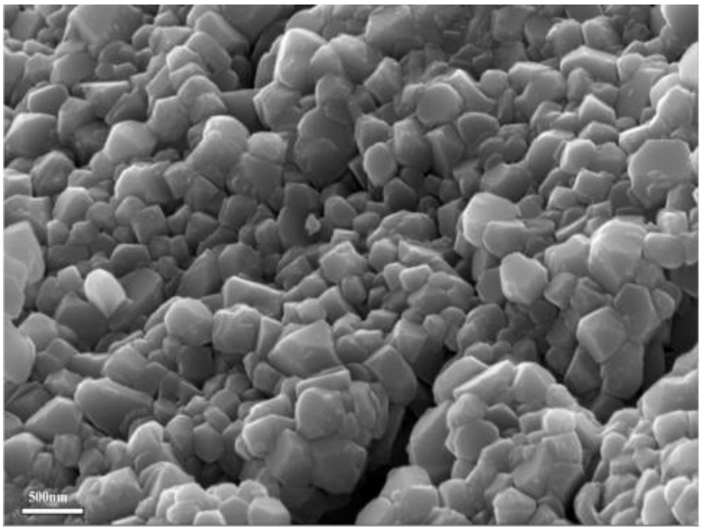

Figure S1. SEM image of LMCNOLF, showing primary crystalline particles with scale bar of 500 nm.

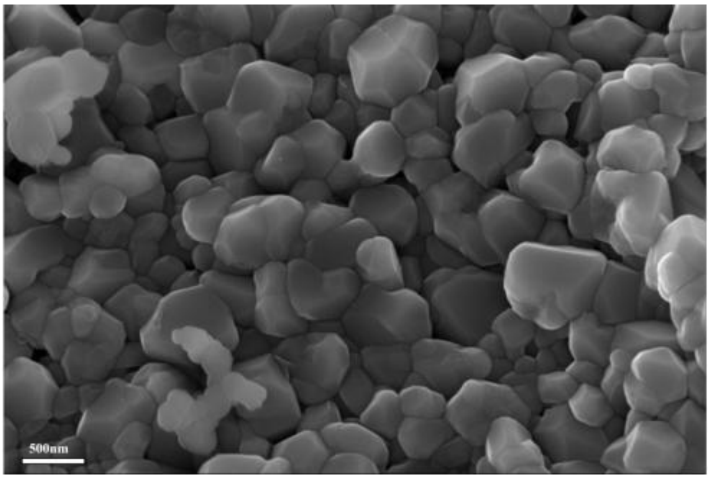

Figure S2. SEM image of LMCNOL material with a scale bar of 500 nm.

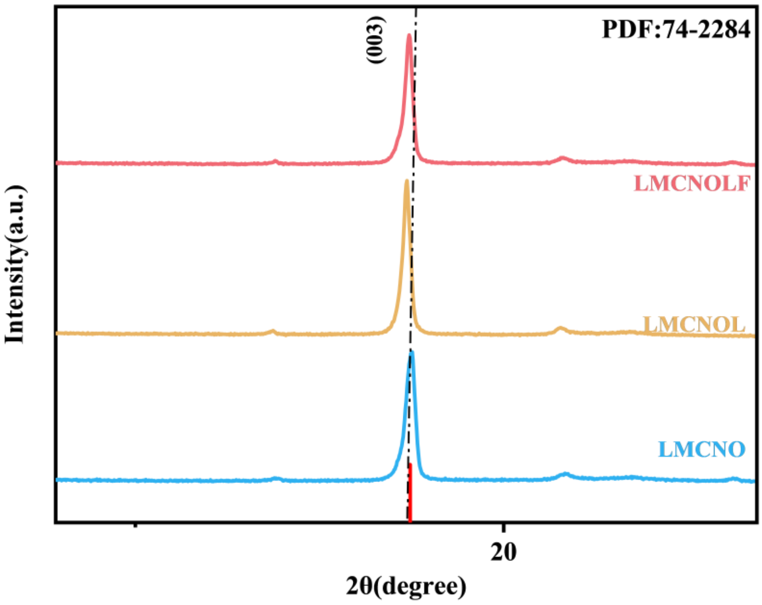

Figure S3. Locally magnified XRD patterns of the (003) diffraction peak for LMCNO, LMCNOL, and LMCNOLF samples (indexed to JCPDS/PDF No. 74-2284), illustrating the peak shift toward lower 2θ angles upon doping.

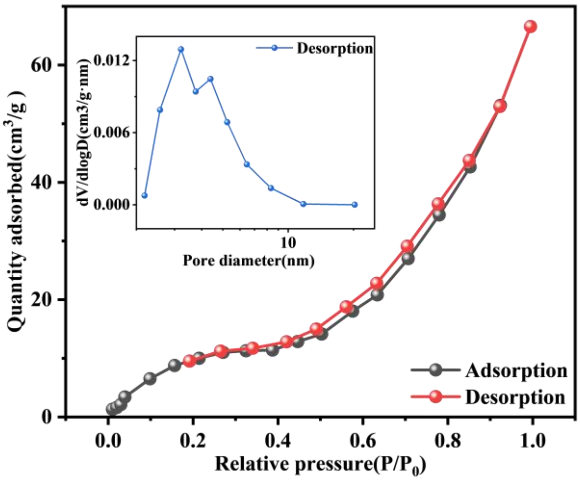

Figure S4. Nitrogen adsorption–desorption isotherms of the synthesized sample. The inset shows the corresponding pore size distribution curve calculated from the desorption branch.

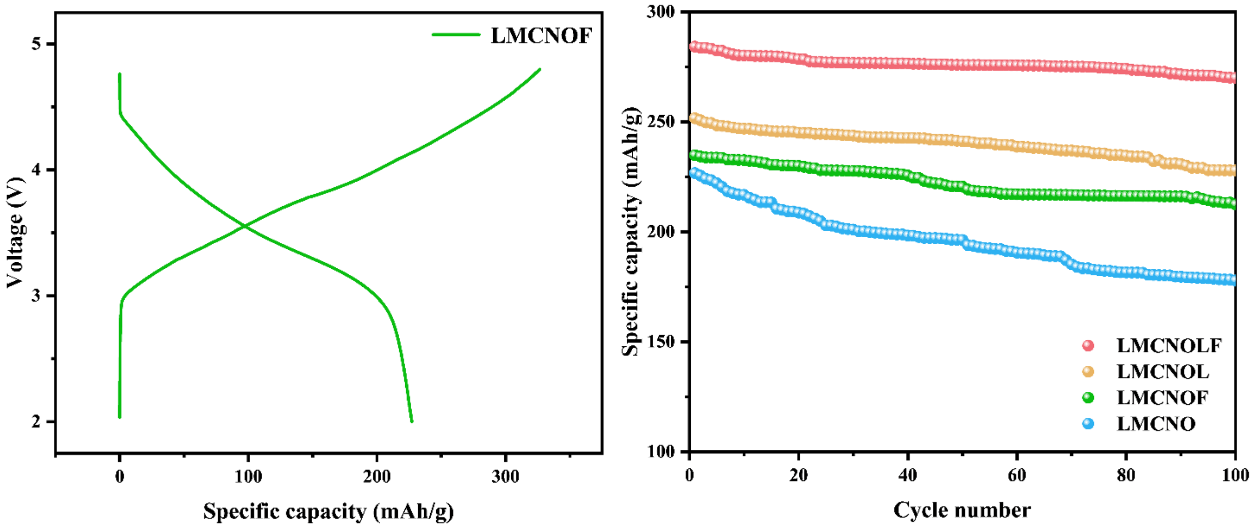

Figure S5. Variation of specific capacity versus cycle number for LMCNO, LMCNOL, LMCNOF and LMCNOLF cathodes over 100 cycles.

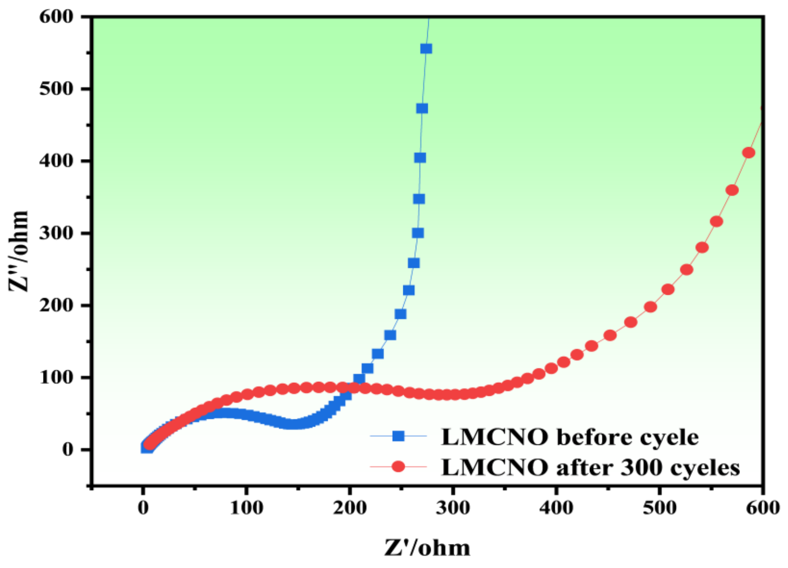

Figure S6. EIS Nyquist spectra of LMCNO before cycling and after 300 cycles.

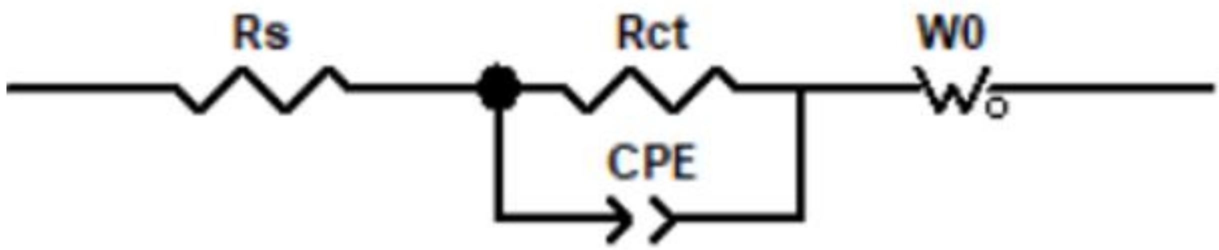

Figure S7. Equivalent electrical circuit model (R_s(CPE-R_{ct})Z_w) used for fitting the electrochemical impedance spectra of the LMCNO, LMCNOL, and LMCNOLF electrodes.

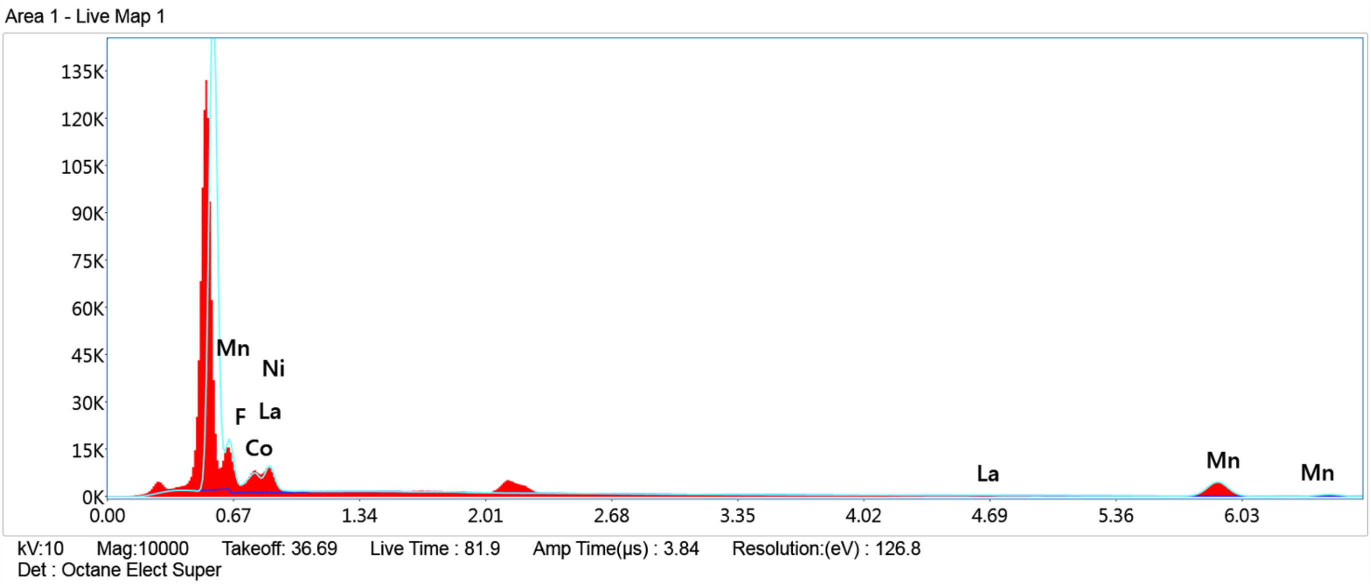

Figure S8. Energy-dispersive X-ray spectroscopy (EDS) spectrum of the LMCNOLF sample, confirming the presence of Mn, Ni, Co, La, and F elements.

**Performance parameter**	**LMCNOLF**	**LMCNOL**	**LMCNO**
**Initial charge specific capacity(10mA g^{-1})**	346 mAh g^{-1}	331 mAh g^{-1}	325 mAh g^{-1}
**Initial discharge specific capacity(10mA g^{-1})**	292 mAh g^{-1}	246 mAh g^{-1}	221 mAh g^{-1}
**Initial Coulombic efficiency**	84%	74%	68%
**Capacity before long-term cycling(100mA g^{-1})**	217 mAh g^{-1}	192 mAh g^{-1}	147 mAh g^{-1}
**Capacity after long-term cycling (100mA g^{-1})**	195 mAh g^{-1}	162 mAh g^{-1}	110 mAh g^{-1}
**Capacity retention after long-term cycling**	89 %	84%	74%

Table S1. Summary of electrochemical performance parameters for the LMCNO, LMCNOL, and LMCNOLF cathode electrodes.
